# Predicting Mutation-Induced Allosteric Changes in Structures and Conformational Ensembles of the ABL Kinase Using AlphaFold2 Adaptations with Alanine Sequence Scanning

**DOI:** 10.3390/ijms251810082

**Published:** 2024-09-19

**Authors:** Nishank Raisinghani, Mohammed Alshahrani, Grace Gupta, Gennady Verkhivker

**Affiliations:** 1 Keck Center for Science and Engineering, Schmid College of Science and Technology, Chapman University, Orange, CA 92866, USA; nishankr@stanford.edu (N.R.); alshahrani@chapman.edu (M.A.); grgupta@chapman.edu (G.G.); 2 Department of Biomedical and Pharmaceutical Sciences, Chapman University School of Pharmacy, Irvine, CA 92618, USA

**Keywords:** protein kinases, molecular mechanism, protein dynamics, allosteric mutations, conformational landscapes, allosteric states, artificial intelligence, structural modeling

## Abstract

Despite the success of AlphaFold2 approaches in predicting single protein structures, these methods showed intrinsic limitations in predicting multiple functional conformations of allosteric proteins and have been challenged to accurately capture the effects of single point mutations that induced significant structural changes. We examined several implementations of AlphaFold2 methods to predict conformational ensembles for state-switching mutants of the ABL kinase. The results revealed that a combination of randomized alanine sequence masking with shallow multiple sequence alignment subsampling can significantly expand the conformational diversity of the predicted structural ensembles and capture shifts in populations of the active and inactive ABL states. Consistent with the NMR experiments, the predicted conformational ensembles for M309L/L320I and M309L/H415P ABL mutants that perturb the regulatory spine networks featured the increased population of the fully closed inactive state. The proposed adaptation of AlphaFold can reproduce the experimentally observed mutation-induced redistributions in the relative populations of the active and inactive ABL states and capture the effects of regulatory mutations on allosteric structural rearrangements of the kinase domain. The ensemble-based network analysis complemented AlphaFold predictions by revealing allosteric hotspots that correspond to state-switching mutational sites which may explain the global effect of regulatory mutations on structural changes between the ABL states. This study suggested that attention-based learning of long-range dependencies between sequence positions in homologous folds and deciphering patterns of allosteric interactions may further augment the predictive abilities of AlphaFold methods for modeling of alternative protein sates, conformational ensembles and mutation-induced structural transformations.

## 1. Introduction

AlphaFold2 (AF2) technology represents a significant leap forward in protein structure modeling, marking a transformative era in structural biology [1,2]. AF2 utilizes evolutionary insights obtained from Multiple Sequence Alignments (MSAs) derived from related protein sequences. It employs a hierarchical transformer architecture equipped with self-attention mechanisms, enabling the identification of long-range dependencies and interactions within protein sequences [1,2]. These self-supervised deep learning models draw inspiration from natural language processing (NLP) architectures, particularly those integrating attention-based and transformer mechanisms. They have proven highly effective in capturing contextual spatial relationships by training on extensive datasets of protein sequences [3,4]. A recent breakthrough in AI-driven protein structure prediction is represented by ESMFold, a series of transformer-based protein language models renowned for their exceptional accuracy in predicting atomic-level protein structures directly from individual protein sequences, thereby eliminating the need for Multiple Sequence Alignments (MSA) [5]. Another innovation, OmegaFold, employs a hybrid approach combining a protein language model with a geometry-guided transformer model [6]. Despite the remarkable achievements of AF2-based methods and self-supervised protein language models in accurately predicting static protein structures, they face significant challenges in their application and broad utility for characterizing conformational dynamics, functional protein ensembles, conformational changes, and allosteric states [7]. Recent studies have highlighted that AF2 methods excel in predicting individual protein structures; however, extending this capability to accurately forecast conformational ensembles and map allosteric landscapes remains a significant challenge [8,9,10,11,12]. The limitations of AF2 methods in predicting multiple protein conformations may arise from a training bias towards experimentally verified, thermodynamically stable structures, and MSAs that predominantly capture evolutionary information used to infer ground protein states. This bias potentially restricts their ability to fully capture the diversity of conformational dynamics. Some studies suggest that AF2 methods face difficulties in expanding their predictive capabilities beyond characterizing functional conformational ensembles and accurately mapping allosteric landscapes [13,14,15,16,17].

Several recent adaptations of the AF2 framework aimed at predicting alternative conformational states of proteins involve strategies such as reducing the depth of the Multiple Sequence Alignment (MSA) to sample only a subset of sequences, resulting in a shallower MSA [18]. This approach is designed to increase the diversity of sequences used in modeling protein structures, potentially enabling the capture of a wider range of alternative conformational states. The SPEACH_AF (Sampling Protein Ensembles and Conformational Heterogeneity with AlphaFold2) approach employs in silico alanine mutagenesis within MSAs to expand the attention network mechanism within AF2, facilitating exploration of distinct patterns of coevolved residues associated with alternative conformations [19]. Another adaptation, the AF-Cluster method, involves MSA subsampling followed by clustering of evolutionarily related or functionally similar sequences. This method allows predictions of alternative protein states and has demonstrated success in identifying previously unknown fold-switched states, which were subsequently validated using NMR analysis [20]. Recent advancements in AF2 pipelines have focused significantly on expanding the range of accessible protein conformations. These adaptations integrate sequence and evolutionary data derived from MSAs, as well as structural insights obtained from templates. This approach has been particularly successful in applications to proteins like kinases and GPCRs [21]. Current developments highlight key challenges in AF2 methodologies and their adaptations. These include accurately capturing functional conformational ensembles, understanding allosteric states, and elucidating the impacts of mutations on both local and global protein conformational changes [22].

Protein kinases are dynamic regulatory switches, constantly transitioning between active and inactive states in a dynamic equilibrium [23,24]. The extensive availability of structural information on protein kinases, various activating and inactivating mutants and a large variety of complexes with ATP-competitive and allosteric inhibitors [25,26] across various functional contexts provides unmatched opportunities to validate and refine AF2 methods. A diverse range of simulation strategies, augmented by Markov state models (MSMs), has been effectively employed to comprehensively explore the free energy landscape of ABL kinase, surpassing the conformations identified through X-ray crystallography [27,28]. However, these pioneering computational studies have also highlighted the inherent challenges and limitations of conventional biophysical simulations in accurately characterizing transient states and describing the kinetics of conformational changes. The enhanced sampling approaches often fail to detect functionally relevant conformations of protein kinases and are unable to accurately map complex allosteric landscapes underlying kinase regulation and activation. Despite the extensive structural knowledge accumulated for protein kinases, experimental atomistic characterizations of functionally relevant transient states have historically been challenging. This is primarily due to the large conformational transformations and short-lived intermediates involved in the kinetics of allosteric shifts. Recent advancements in NMR spectroscopy have provided an atomistic view of the energy landscape governing allosteric regulation in ABL kinase. These studies have elucidated how structural elements work synergistically to create a complex allosteric mechanism that facilitates the transition between distinct allosteric states [29]. In another pioneering study by the same research group, NMR chemical exchange saturation transfer (CEST) experiments were employed to characterize the structure of unbound ABL kinase in the active state. This approach enabled the identification and structural characterization of two short-lived inactive conformations which are intrinsic to the kinase domain and differ significantly in critical functional regions [30]. These findings showed that ABL kinase dynamically switches between active and distinct inactive states within its conformational ensemble, a process regulated by mutants, ligands, post-translational modifications, and inhibitors to modulate kinase activity and function [30]. Atomistic molecular dynamics (MD) simulations and Markov State kinetic modeling have further contributed to understanding the dynamics of structural changes between these ABL kinase conformational states [31].

Recent investigations have explored the potential of AF2 methodologies in predicting conformational states in protein kinases. One study utilized AF2-based modeling to assess 437 human protein kinases in their active form, employing shallow Multiple Sequence Alignments (MSAs) derived from orthologs and close homologs of the target protein. This investigation underscored the robustness of AF2 methods, where models selected for each kinase based on prediction confidence scores of activation loop residues closely matched substrate-bound experimental structures [32]. Another study investigated AF2′s capability to predict kinase structures in various conformations across different depths of MSAs revealing that lower MSA depths enabled more efficient exploration of alternative kinase conformations [33]. Several different adaptations of the MSA subsampling approach were systematically explored to characterize the conformational distributions of the ABL kinase domain using AF2 methodologies. This research specifically examined the method’s ability to predict the effects of mutations that either decrease or increase the population of the ground state. The findings highlighted the potential of AF2 methods while also pointing out significant limitations in predicting the effects of mutations on shifting the equilibrium between active and inactive states. Notably, the predicted effects often diverged from experimental observations regarding changes in ground state population induced by mutations [34]. We recently introduced a novel adaptation of AF2, combining randomized alanine sequence scanning across the entire protein sequence or specific functional regions with MSA subsampling. This approach provided a thorough characterization of the conformational ensembles of the ABL kinase in both active and inactive states [35]. However, the primary challenges of AF2 adaptations are associated with accurately predicting functional conformations and determining the relative populations of distinct allosteric states rather than merely increasing the diversity of predicted ensembles.

While AF2 is typically robust in predicting single protein structures, these methods are limited when it comes to predicting multiple functional conformations of allosteric proteins or accurately capturing the effects of single-point mutations, which can lead to significant structural changes. When mutations lead to large changes in the native structure, the performance of AF2 predictions may be compromised [36,37,38]. A recent analysis of AF2 methods for predicting effects of point mutations showed that functionally relevant structural changes in the mutational models can be obtained when mutations are introduced in the entire MSA as compared to only the input sequence [39]. The latest studies demonstrated that AF2 may be able to predict single mutation effects with moderate structural changes [40]. Moreover, AF2-predicted structures can encode information on stability and destabilizing effects of single mutations that do not disrupt protein structure [41]. As the number of possible mutations far exceeds the data points used to train AF2 methods, it remains challenging to accurately capture the full range of mutation effects. This challenge is further compounded by the fact that critical changes in stability often stem from subtle structural alterations whereas many important disease-associated mutations are allosteric and could induce large structural transformations and ensemble redistribution.

In this study, we advance our AF2 adaptation by incorporating targeted alanine masking across critical functional regions of the ABL kinase sequence, alongside MSA subsampling. We conduct a comparative analysis between this approach and AF2 with random alanine sequence scanning, focusing on predicting structures, conformational ensembles, and populations for ABL active and inactive wild-type (WT) states as well as several double ABL mutants that are known to affect the equilibrium between the active ABL form and the inactive states. We show that combining targeted alanine sequence masking with shallow MSA subsampling significantly enhances the diversity of predicted structural ensembles. This approach successfully captures populations of both the active and fully inactive I2 states. Our analysis also underscores the inherent challenges in accurately predicting mutation-induced changes in the relative populations of ABL states using AF2. The predictions tend to be biased towards the ground state of ABL, highlighting limitations in capturing dynamic equilibrium shifts induced by mutations. We also perform a network-based analysis of ABL structures and AF2-generated ensembles. This analysis suggests that functional ABL mutations can directly influence or be structurally proximal to high network centrality positions that can be associated with allosteric hotspots mediating long-range interactions. We argue that integrating specific coevolutionary signals and attention-based learning of allosteric couplings across homologous folds may enhance the predictive capabilities of AF2-based methods in future applications.

## 2. Results and Discussion

### 2.1. Predicting Conformational Ensembles of the ABL Kinase Mutants Using Shallow MSA Subsampling AF2: Emerging Bias towards the Active ABL Form

**Figure 1:**
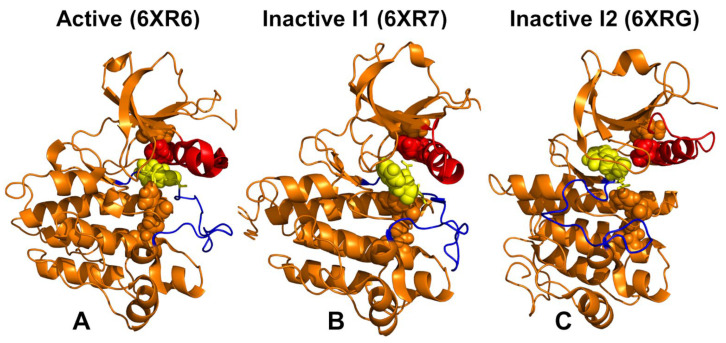
The thermodynamically stable fully active ground state of the ABL kinase domain (pdb id 6XR6) (**A**), the inactive state I_1_ (pdb id 6XR7) (**B**) and the closed inactive state I_2_ (pdb id 6XRG) (**C**). The ABL structures are shown in orange ribbons. The αC-helix (residues 291–311) is shown in red ribbons, the A-loop (residues 398–421) is shown in blue ribbons and the 400-DFG-402 motif is shown in yellow sticks. The R-spine residues M309, L320, H380, F401 and D440 are also shown in spheres. The experimental ABL structures are NMR ensembles with 20 structures each. The presented structures for each of the ABL states correspond to the first conformation in the respective NMR ensemble.

The N-lobe of ABL is composed of a five-stranded antiparallel β-sheet and a single αC-helix (residues 291–311) while the C-lobe (residues 341–531) is mainly helical and encompasses the peptide substrate-binding site. Conformational transitions between kinase states are orchestrated by three conserved structural motifs in the catalytic domain: the αC-helix, the 400-DFG-402 motif in ABL (DFG-in, active; DFG-out, inactive), and the activation loop (A-loop open, active; A-loop closed, inactive) [23,24]. The conserved His-Arg-Asp (HRD) motif in the catalytic loop and the DFG motif are coupled with the αC-helix to form conserved intramolecular networks termed regulatory spine (R-spine) and catalytic spine (C-spine). The NMR ensemble of the active conformations (pdb id 6XR6) is characterized by the “αC-in” position and stable DFG-in orientation (Figure 1A), while in the inactive I_1_ state (pdb id 6XR7), the αC helix moves to the intermediate αC-out position and the DFG motif is flipped 180°, with respect to the active conformation (Figure 1). In the inactive I_2_ state, the regulatory DFG motif assumes a distinct “out” conformation and the A-loop swings to a fully closed conformation (Figure 1C).

Structural analysis and alignment of the three ABL states further illustrates that the inactive ABL states are very different as the regulatory DFG motif adopts distinct “DFG-out” arrangements in the I_1_ and I_2_ states (Supporting Information, Figure S1). Moreover, structural differences between ABL states are particularly evident in the A-loop and the αC helix that undergo significant rearrangements during conformational transitions between the inactive and active states. In particular, the αC helix moves from its active “αC-in” position to the intermediate position in the I_1_ state and to the “αC-out” inactive position in the I_2_ state (Supporting Information, Figure S1B). In the fully inactive conformation, the key structural elements, A-loop, DFG motif, and αC helix, adopt conformations that are not compatible with either substrate binding or ATP binding or hydrolysis. The R-spine subnetwork (residues M309, L320, H380, F401 and D440 is fully assembled in the active ABL kinase but becomes partially decoupled in the inactive state I_1_ and fully disassembled in the inactive state I_2_ (Supporting Information, Figure S1). The C-spine is comprised of hydrophobic residues (V275, A288, L342, C388, L389, V336, S457, and I451) that connect the kinase lobes anchoring catalytically important sites to the C-terminus of the αF-helix (residues 436–453).

The NMR ensemble of the active conformation conforms to the thermodynamically dominant state with some appreciable degree of conformational plasticity in the A-loop and the αC helix (Supporting Information, Figure S2A). Notably, all conformations from the active ensemble are characterized by the “αC-in” position and stable DFG-in orientation (Supporting Information, Figure S2A). In the NMR ensemble of the inactive I_1_ form the DFG motif is flipped 180°, with respect to the active conformation, but the A-loop remains in an open and highly heterogeneous conformation similar to the active conformation (Supporting Information, Figure S2B). The analysis of the NMR ensembles showed a contrast between the heterogeneous I_1_ ensemble (Supporting Information, Figure S2B) and a more restricted inactive I_2_ ensemble which is different structurally and dynamically (Supporting Information, Figure S2C).

We also used NMR conformational ensembles to probe dynamic cross-correlations between pairs of residues in different ABL states (Supporting Information, Figure S2D–F). The dynamic cross-correlation (DCC) analysis produces an NxN heatmap, where N is the number of alpha carbon atoms in the system, and each element corresponds to the dynamic cross-correlation between pairs of atoms i and j. The DCC between the i and j atoms is defined by Equation (1) where $\Delta r_{i}$ is the displacement from the average position of atom i and 〈⬚〉 is the average over the ensemble.

(1) $$C_{ij}=\frac{<\Delta r_{i}\cdot \Delta r_{j}>}{\sqrt{<\Delta r_{i}^{2}>}\cdot \sqrt{<\Delta r_{j}^{2}>}}$$

The results can be compared with a similar analysis performed in our earlier study where we utilized MD simulations of the active and inactive ABL states [31]. As may be expected, NMR ensembles (Supporting Information, Figure S2D–F) and MD trajectories (Figure 2 in [31]) yield a generally similar pattern of dynamic couplings. In the active ABL ensemble, we observed strong positive correlations between the catalytic core, ATP site and C-lobe regions, particularly between the αF-helix (residues 436–453 in the original structure), the αD-helix (residues 341–348), and the A-loop (residues 398–421) (Supporting Information, Figure S2D). Hence, the residue correlation pattern in the NMR ensemble of active conformations pointed to long-range couplings between its lobes. Perhaps not surprisingly, our previous analysis based on MD simulations of the active ABL form produced even stronger positive dynamic couplings, signaling presence of allosteric interactions that link the ATP binding site and the substrate binding regions. A direct dynamic correlation analysis of the NMR ensemble, which is more heterogeneous than the MD-generated ensemble in [31], resulted in only a slightly more diffused coupling pattern, supporting the notion of positive allostery in the active state. Similar to the analysis of MD trajectories [31], the dynamic correlation analysis of the NMR ensemble for the inactive state I_1_ displayed only moderately weakened positive couplings between functional regions and a narrower network of correlated residues (Supporting Information, Figure S2E). These observations are consistent with the increased heterogeneity of the inactive state I_1_ that nonetheless retains the open active A-loop conformation. The further weakening of the positive long-range dynamic couplings was observed in the NMR ensemble of the inactive state I_2_ (Supporting Information, Figure S2F) which is consistent with the analysis of MD trajectories [31]. In general, the cross-correlation maps of residue fluctuations in the ABL catalytic domain were indicative of more cooperative allosteric interactions in the active state as compared to narrower patterns of correlated residues in the inactive states. Hence, the NMR ensembles and MD-generated trajectories resulted in a consistent dynamic pattern of dynamic cross-correlations, showing a progressive reduction of long-range allosteric interactions as ABL transitions from the active to a fully inactive form.

**Figure 2:**
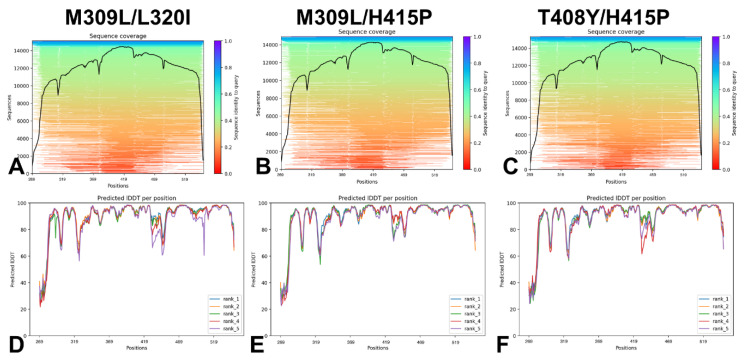
The statistical analysis of the AF2 experiments with shallow MSA subsampling for the ABL mutants: M309L/L320I mutant (**A**), M309L/H415P mutant (**B**), H415P/T408Y mutant (**C**), and a heatmap representation of the MSA indicates all sequences mapped to the input sequences. The color scale points to the identity score, and sequences are ordered from top (largest identity) to bottom (lowest identity). White regions are not covered, which occurs with sub-sequence entries in the database. The black line qualifies the relative coverage of the sequence with respect to the total number of aligned sequences. The residue-based pLDDT profiles of the top five ranked models from the AF2 experiments with shallow MSA subsampling for the ABL mutants: M309L/L320I mutant (**D**), M309L/H415P mutant (**E**), and H415P/T408Y mutant (**F**).

NMR studies also reported the structure of the ground state (with a population 88%) of the isolated Abl kinase domain in solution in which the ABL kinase domain adopts a catalytically active conformation with the A-loop adopting an open conformation and the DFG motif and αC helix both in the “in” state [30]. These investigations determined that M309L mutation in the R-spine increases the population of I_1_ and I_2_ to 10% and 35%, respectively, while double M309L/L320I substitution switches the ensemble towards the I_2_ state (~82%) thus deactivating the kinase [30]. The ABL mutant M309L/H415P shifts the equilibrium towards I_1_ without eliciting other structural changes, while T408Y/H415P, increases the population of the I_2_ state [30]. We employed several different AF2 adaptations, including shallow MSA subsampling approach and randomized alanine sequence scanning [35], to predict structures and conformational ensembles for double ABL mutants M309L/L320I, M309L/H415P, and T408Y/H415P that have unique effects on the conformational equilibrium and can induce large conformational transformations between the active and inactive ABL ensembles. First, we analyzed the AF2 predictions for ABL mutants using the shallow MSA approach. The AF2-produeced MSAs are summarized as a heatmap indicating all sequences mapped to the input sequences (Figure 2A–C).

The relative coverage of the sequence with respect to the total number of aligned sequences is shown indicating the reduced sequence identity to query for the highly flexible N-lobe regions, while a statistically significant sequence coverage was seen for the kinase domain core and C-lobe regions (residues 341–531) (Figure 2A–C). The shallow MSA subsampling predictions converged predominantly to the active ABL conformation for all examined ABL mutants, showing high confidence pLDDT values (Figure 2D–F). Most of the ABL kinase core regions featured pLDDT ~80–100, while the A-loop (residues 395–421) displayed moderately reduced pLDDT values ~65–85 and low pLDDT values only for highly flexible N-lobe regions (Figure 2D–F). Some of the low confidence pLDDT values corresponded to disordered N-terminal residues that were revealed in the NMR ensembles.

**Figure 3:**
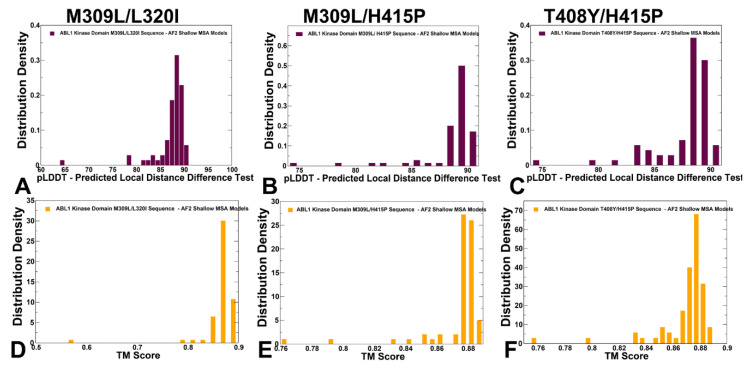
The analysis of AF2 predictions of the conformational ensembles for ABL mutants using the shallow MSA subsampling approach. The density distributions of the pLDDT estimates for the predicted models in conformational ensembles of the M309L/L320I mutant (**A**), M309L/H415P mutant (**B**), and H415P/T408Y mutant (**C**). The pLDDT structural model estimate of the prediction confidence is on a scale from 0 to 100. The distribution density of TM-scores for the predicted ABL mutant conformations with respect to the experimental active ABL state (orange filled bars) for M309L/L320I mutant (**D**), M309L/H415P mutant (**E**), and H415P/T408Y mutant (**F**). The TM-scores are computed with respect to the first conformation in the respective NMR ensembles of the active form (pdb id 6XR6), the inactive form I_1_ (pdb id 6XR7) and the inactive form I_2_ (pdb id 6XRG).

To gain a quantitative insight into the AF2 predictions, we analyzed the pLDDT density distribution for the predicted conformational ensembles of the ABL mutants (Figure 3A–C). The dominant peaks at pLDDT ~85–90 and several minor peaks for pLDDT~70–75 are present for all mutants, indicative of similar conformational ensembles produced by the shallow MSA approach for all mutants (Figure 3A–C). Instructively, shallow MSA subsampling predictions reflected conformational heterogeneity of the active state that is exemplified in appreciable fluctuations of the open A-loop conformations. The TM-scores of the AF2-predicted conformations showed narrow peaks of TM values ~0.85–0.88 with respect to the active ABL conformation, indicating that the overwhelming majority of the predicted conformations are structurally similar to the active ABL form (Figure 3D–F).

**Figure 4:**
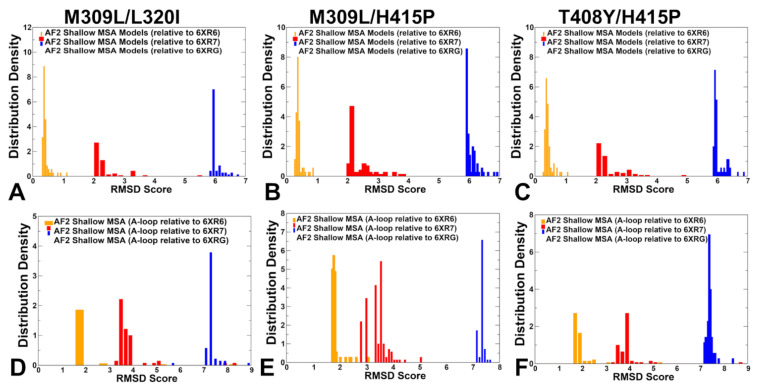
The analysis of AF2 predictions of the conformational ensembles for ABL mutants using the shallow MSA subsampling approach. The density distributions of the RMSD values for predicted conformational ensembles of the M309L/L320I mutant (**A**), M309L/H415P mutant (**B**), and H415P/T408Y mutant (**C**). The distribution density of RMSD scores for the AF2-predicted conformations are shown relative to the active ABL state (orange filled bars) and the inactive states I_1_ (red filled bars) and I_2_ (blue filled bars). The density distributions of the RMSD values for the A-loop (residues 395–421) in the predicted ensembles are shown for M309L/L320I (**D**), M309L/H415P (**E**), and H415P/T408Y (**F**). The experimental ABL structures are NMR ensembles with 20 structures each. The RMSD values are computed with respect to the first conformation in the respective NMR ensembles of the active form (pdb id 6XR6), the inactive form I_1_ (pdb id 6XR7) and the inactive form I_2_ (pdb id 6XRG).

We also computed the root mean square deviations (RMSD) distributions of the predicted ensembles with respect to the active and inactive experimental structures (Figure 4A–C). The RMSD densities showed a clear separation between the RMSDs computed with respect to different ABL states, particularly highlighting the dominant peaks for RMSDs < 1.0 Å relative to the active form (Figure 4A–C). We observed contributions of conformations that are similar to the intermediate inactive I_1_ form (RMSD~2.0Å) but these similarities largely reflect the intermediate nature of the inactive I_1_ state in which the A-loop remains in an open conformation and the N-terminal part of the A-loop is similar in the active and I_1_ states. However, the NMR ensemble for the I_1_ form features the DFG motif being flipped 180° with respect to the active conformation and adopting a DFG-out position. To characterize conformational changes in the A-loop, we also computed the RMSD densities for the A-loop residues only (Figure 4D–F). This analysis confirmed that the majority of the ensemble conformations conforms to the active ABL form, revealing larger fluctuations of the active A-loop and emergence of the conformations with an A-loop similar to the inactive I_1_ state (RMSD~2.8–3.5 Å) for the M309L/L320I and M309L/H415P double mutants (Figure 4D,E).

**Figure 5:**
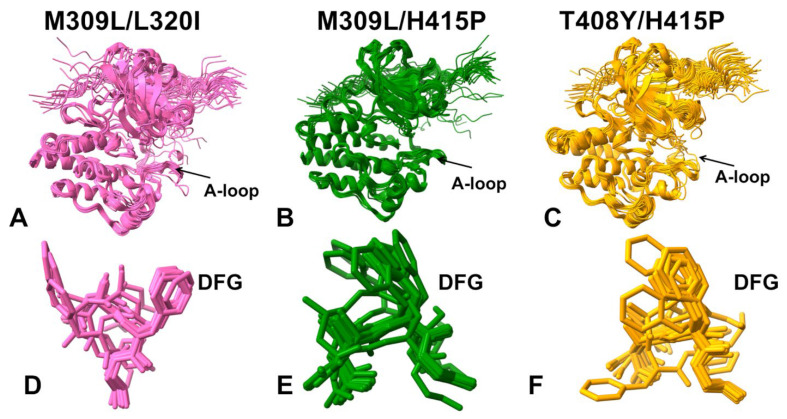
Structural alignment of the AF2-predicted ensembles using the shallow MSA subsampling approach. Structural overlay of the kinase conformations from the AF2-predicted conformational ensemble for the M309L/L320I mutant (**A**), M309L/H415P mutant (**B**), and T408Y/H415P mutant (**C**). Structural overlay of the regulatory DFG motif conformations from the predicted conformational ensembles of the M309L/L320I mutant (**D**), M309L/H415P mutant (**E**), and H415P/T408Y mutant (**F**).

Structural mapping of the conformational ensembles showed moderate perturbations of the A-loop but the M309L/L320I mutation caused more significant fluctuations without changing the extended open arrangement of the A-loop (Figure 5A–C). The alignment of the DFG conformations showed that both DFG-in and DFG-out with F401 pointing upwards can be seen for the M309L/L320I double mutant (Figure 5D). For the M309L/H415P (Figure 5E) and T408T/H415P mutant (Figure 5F), the ensemble is primarily dominated by DFG-in conformation, and it also samples intermediate DFG-out positions. Interestingly, this is consistent with the fact that the H415P mutation can destabilize the I_2_ state and increase stabilization of the I_1_ state (with intermediate DFG-in/out conformations) [30]. The shallow MSA AF2 predictions may reflect the increased heterogeneity of the A-loop induced by mutations in the A-loop (T408Y and H415P), in some conformations, but the overall predicted ensembles are largely dominated by the active ABL states. Hence, the AF2 predictions using shallow MSA depth adaptation revealed only a limited variability of the regulatory DFG-in conformation that remained confined to its active form. Although subsampling of MSA may increase conformational heterogeneity around the ground active ABL state, this approach cannot readily generate inactive ABL conformations that become more dominant in the ABL double mutant M309L/L320I.

### 2.2. Alanine Sequence Scanning Combined with Shallow MSA Subsampling Can Detect Population Shifts in State-Switching ABL Mutants

**Figure 6:**
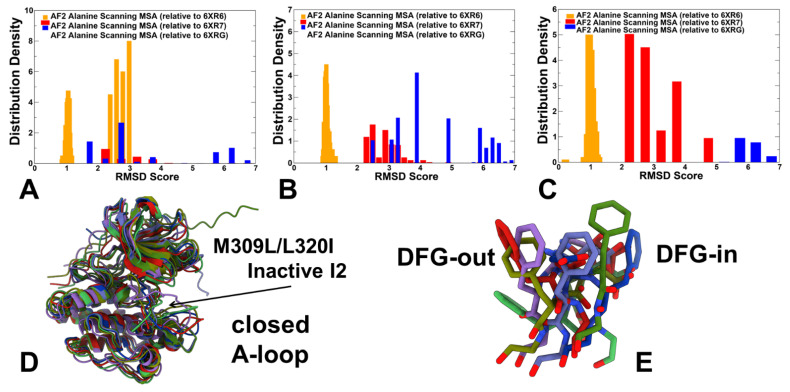
The analysis of AF2 predictions using the randomized alanine sequence scanning approach. The distribution density of RMSD scores for the AF2-predicted ABL conformational ensembles of the M309L/L320I mutant (**A**), M309L/H415P mutant (**B**), and H415P/T408Y mutant (**C**). The distribution density of RMSD scores for the AF2-predicted conformational ensembles are shown relative to the active ABL state (orange filled bars) and the inactive states I_1_ (red filled bars) and I_2_ (blue filled bars). The RMSD values are computed with respect to the first conformation in the respective NMR ensembles of active form (pdb id 6XR6), inactive form I_1_ (pdb id 6XR7) and inactive form I_2_ (pdb id 6XRG). Structural alignment of the AF2-predicted conformations for the M309L/L320I mutant that are close to the inactive I_2_ state (RMSD < 1.5 Å). (**D**) The predicted conformations are obtained using the randomized alanine sequence scanning approach combined with shallow MSA subsampling in AF2. The A-loop conformation adopts a full closed conformation as in the inactive I_2_ experimental structure. (**E**) Structural alignment of the DFG motifs that adopt a number of intermediate DFG-out positions in the predicted inactive conformations.

We employed recently developed randomized alanine scanning adaptation of the AF2 methodology in which the algorithm operates first on the pool of sequences and iterates through each amino acid in the native sequence to randomly substitute 5–15% of the residues with alanine, thus emulating random alanine mutagenesis. To probe differences between randomized alanine scanning of the entire protein sequence and targeted random scanning of specific kinase regions, we also examined several variations of this approach with targeted alanine masking of the ABL sequence space. Random alanine masking of sequence positions in regions involved in conformational changes included the A-loop (residues 398–421) and the αC-helix (residues 291–311). We analyzed the RMSD values computed for the predicted ABL structure with respect to the active and inactive experimental ABL structures (Figure 6). The results showed a significant overlap in the distributions for M309L/L320I (Figure 6A) and M309L/H415P (Figure 6B). Accordingly, the predictions produced a significant functionally relevant ensemble of the heterogeneous inactive I_1_ conformations in these mutants, particularly for M309L/H415P. This is consistent with the experimentally observed shifts towards the inactive state for these ABL mutants [30]. These findings can be contrasted with the predictions using the shallow MSA approach where the vast majority of the generated conformations for all ABL mutants converged towards the active ABL form and could not detect the experimentally observed switching to the inactive I_2_ form. We also illustrated the predicted inactive I_2_ conformations for the ABL state-switching mutant M309L/L320I (Figure 6D,E). It should be noted that the population of these inactive states remains relatively diverse, but they share a flipped A-loop conformation that switches from the open to closed conformation. The predicted conformations also featured some variability of the DFG-out position (Figure 6E).

**Figure 7:**
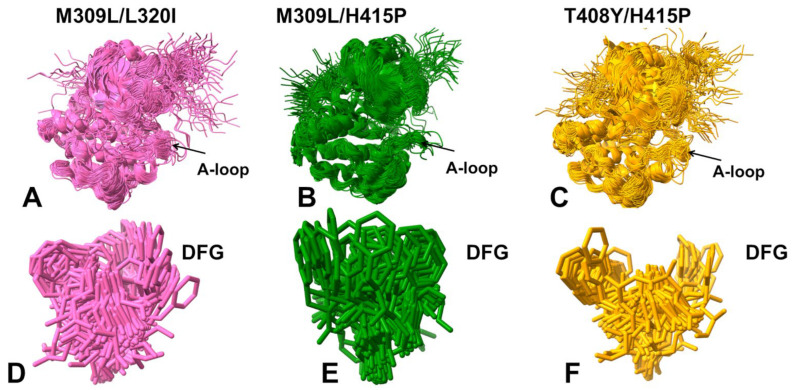
Structural alignment of the conformational ensembles in the ABL mutants obtained with AF2 adaptation based on alanine sequence scanning. Structural overlay of the AF2-predicted conformational ensemble and DFG motifs are shown for the M309L/L320I mutant (**A**), M309L/H415P mutant (**B**), and H415P/T408Y mutant (**C**). Structural superpositions of the sampled DFG conformations in the predicted ensembles for the respective ABL mutants are shown in panels (**D**–**F**).

Structural mapping of the predicted ABL conformations illustrated a considerably increased conformational heterogeneity of structural ensembles, also highlighting important differences between ABL mutants (Figure 7). Indeed, we observed significant variations of the A-loop that sampled both the open (active) and closed (inactive) conformations in M309L/L320I (Figure 7A) and M309L/H415P (Figure 7B). The variability of the open A-loop conformations was seen for the double mutant T408Y/H415P signaling that the predicted ensemble sampled the active (αC-in, DFG-in, A-loop open) and intermediate inactive I_1_ ABL states (αC-in, DFG-in/out, highly flexible open A-loop) (Figure 7C). By projecting the AF2-produced DFG conformations, the functionally relevant positional variability of the DFG motif between active DFG-in position and inactive DFG-out conformations can be seen (Figure 7D–F). The variability of the DFG motif is particularly exemplified by the observed movements of the F401 residue that samples a large number of intermediate states between DFG-in and DFG-out flipped by 180° for all ABL mutants, In particular, we observed extensive sampling of the DFG-in and DFG-out positions for the M309L/L320I mutant (Figure 7D) and M309L/H415P (Figure 7E) that shift populations of the ABL from the active to inactive I_2_ form [30].

### 2.3. Principal Component Analysis and Comparison of the NMR Ensembles and AF2-Derived Conformational Ensembles for ABL States and State-Switching ABL Mutants

**Figure 8:**
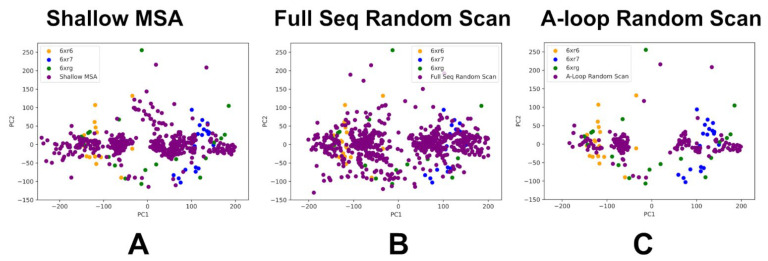
PCA plots for the merged ensemble of the ABL WT consisting of NMR ensembles of the active and inactive ABL state combined with AF2-generated ensembles of the ABL WT using three different AF2 adaptations. (**A**) PCA of the merged ABL WT ensemble consisting of the experimentally determined NMR ensemble and shallow MSA AF2-generated ensemble projected on the common reference coordinate system. (**B**) PCA of the merged ABL WT ensemble consisting of the experimentally determined NMR ensemble and full sequence scan-based AF2-generated ensemble projected on the common reference coordinate system. (**C**) PCA of the merged ABL WT ensemble consisting of the experimentally determined NMR ensemble and A-loop random scan-based AF2-generated ensemble projected on the common reference coordinate system. For all three panels (**A**–**C**), the PCA projection for the NMR ABL active state (6XR6) is shown in green-filled circles, for NMR of the inactive I_1_, in blue-filled circles, and the inactive state I_2_, in red-filled circles. PCA projection for the AF2-generated ensemble is shown in purple-filled circles.

Principal Component Analysis (PCA) was carried out first for the ABL WT by merging the NMR ensemble structures for both active and inactive states together with AF2-generated ensembles (Figure 8). We compared the dynamics distribution of the ABL states and PCAs obtained from NMR ensembles and AF2 predictions. To enable this comparison, AF2 predictions of the ABL WT kinase domain were conducted using the shallow MSA approach (Figure 8A), full sequence randomized scanning method (Figure 8B) and a variation of scanning approach with targeted alanine masking of the A-loop (residues 398–421) positions that facilitate conformational changes between the ABL forms (Figure 8C).

PCA of the merged ABL WT ensemble allowed us to highlight and directly compare differences between the predicted and experimental ensembles through projection on a common reference coordinate system (Figure 8). PCA projections of the NMR ensembles for the ABL states revealed unique structural signatures of these functional ABL forms that have only moderate overlaps in the scatter plot of the two principal components. In particular, PCA plots showed separation between the active ABL ensemble and the NMR ensembles of the inactive I_1_ and I_2_ states. At the same time, PCA plots also revealed a noticeable overlap in the projection map of the two principal components for the inactive states, which may reflect common signatures of the NMR ensembles for the inactive states in which αC helix moves to the αC-out position and the DFG motif is flipped 180°, with respect to the active conformation (Figure 8). Interestingly, the PCA plot also revealed that projection of the NMR ensemble on the two principal components showed a broader coverage and separation of the thermodynamically stable active form and fully closed inactive I_2_ state, while the distribution for the inactive I_1_ ensemble is fairly localized and narrow (Figure 8A).

For PCA comparison, we used AF2 ensembles produced by three different AF2 adaptations: shallow MSA (Figure 8A), full sequence random scanning (Figure 8B) and targeted alanine masking across the A-loop of the ABL kinase domain (Figure 8C). The AF2-generated models obtained with shallow MSA subsampling and randomized alanine scanning adaptation of AF2 were first processed to exclude models featuring pLDDT values < 70. The results suggest that the AF2 ensembles generated using the randomized alanine scanning method (Figure 8B) yield a functionally significant coverage of the conformational landscape for ABL WT protein. Moreover, it is evident that this approach can provide a markedly improved sampling as compared to the shallow MSA adaptation of AF2. Of particular interest are significant overlaps with the distributions of both NMR-derived active and inactive ABL states (Figure 8B).

**Figure 9:**
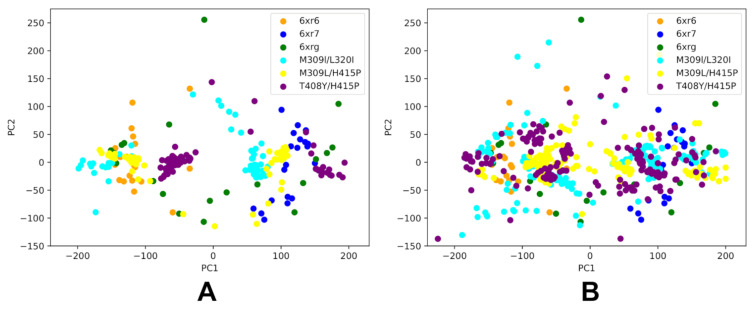
PCA for the merged ensemble consisting of NMR ensembles of the active and inactive ABL state combined with AF2-generated ensembles for three double ABL mutants. The merged ensembles are projected on principal components of the ABL WT that serve a common reference coordinate system. (**A**) PCA plot of the merged ensemble consisting of the experimentally determined NMR ensemble and shallow MSA AF2-generated ensembles of M309L/L320I, M309L/H415P, and H415P/T408Y mutants. (**B**) PCA plot of the merged ensemble consisting of the experimentally determined NMR ensemble and full sequence scan-based AF2-generated ensembles of M309L/L320I, M309L/H415P, and T408Y/H415P mutants. PCA projection for the NMR ABL active state (6XR6) is shown in green-filled circles, the NMR of the inactive I_1_ is shown in blue-filled circles and the inactive state I_2_ is shown in red-filled circles). PCA projection maps for the AF2-generated ensembles for the M309L/L320I mutant are shown in light blue-filled circles, for the M309L/H415P mutant they are shown in yellow-colored filled circles, and for the T408Y/H415P mutant they are shown in purple-filled circles.

We followed with a comparison of PCA maps for conformational ensembles of the ABL double mutants produced by the shallow MSA approach (Figure 9A) and randomized alanine scanning (Figure 9B). These PCA plots were constructed using projection of the merged ensembles on common principal components of the ABL WT derived from the experimental NMR ensembles. Importantly, the experiments showed that for example, the ABL mutant M309L/H415P shifts the equilibrium towards I_1_ while mutants M309L/L320I and T408Y/H415P can increase the population of the I_2_ state [30]. We analyzed PCA distributions for AF2-generated ensembles to examine whether the computationally predicted ensembles can sample both active and inactive ABL forms, possibly capturing the observed population shifts between the ABL states. The AF2-predicted ensembles using the shallow MSA approach are confined to some regions of the conformational space that are mostly associated with the active form and are different from PCA maps of the NMR ensembles for the inactive ABL states. In the case of M309L/H415P, the PCA map of the AF2 ensemble obtained with shallow MSA is notably different from PCA projection of the experimental NMR ensemble (Figure 9A). In contrast, the results revealed that the conformational ensembles generated using random sequence scanning AF2 adaptation yield considerably more diverse spectrum of conformations and enable more adequate sampling of both active and inactive ABL states (Figure 9B). In particular, with this approach, the distributions of sampled conformations for M309L/L320I and T408Y/H415P mutants overlap significantly with the PCA maps of NMR ensembles for the inactive ABL states (Figure 9B). Hence, the AF2-generated ensembles for these state-switching ABL mutants can sample both the inactive I_1_ and I_2_ states. The predictions are consistent with the experimental data showing that state-switching mutants M309L/L320I and T408Y/H415P can alter the equilibrium by moving away from the active state and populate the fully closed inactive I_2_ state [30]. Although the computational predictions using random sequence scanning adaptation of AF2 cannot quantitatively reproduce the observed population shift ratios between the active and inactive I_2_ states, our results suggested that AF2-generated ensembles for state-switching ABL mutants capture the increased diversity and enable productive sampling of hidden allosteric states of ABL.

Together with our previous analyses, the results demonstrate the ability of the proposed approach to capture physically significant population shifts and produce distinct functional conformational clusters that are associated with the active and inactive ABL states. Despite certain limitations in accurately reproducing the ensembles of hidden states and precisely quantifying the changes in relative populations of the active and inactive states, we showed that a combination of alanine sequence masking with MSA construction and shallow MSA subsampling can capture hidden inactive states that become more stable in some of the ABL mutants while avoiding any misfolded predictions.

### 2.4. Network Analysis of the AF2 Conformational Ensembles for ABL Mutants: Mutational Sites Target Predicted Allosteric Hotspots and Induce State-Specific Allosteric Networks

**Figure 10:**
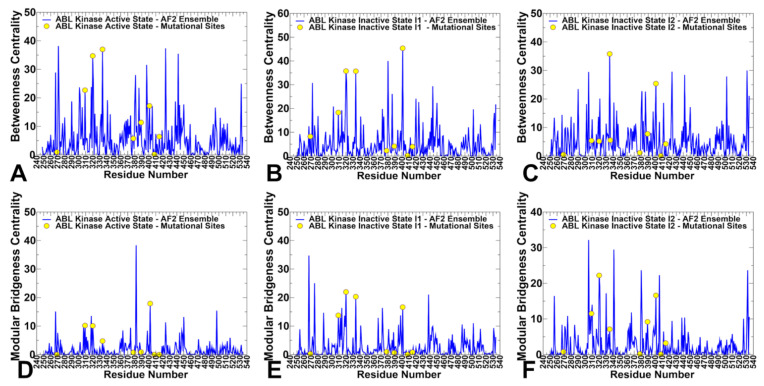
The ensemble-averaged residue betweenness centrality and bridgeness centrality profiles are obtained using conformational ensembles generated by alanine scanning AF2 adaptation for the ABL mutants. The betweenness centrality in the active ABL structure (**A**), inactive I_1_ state (**B**) and inactive I_2_ state (**C**). The bridgeness centrality profiles are shown in the active ABL structure (**D**), inactive I_1_ state (**E**) and inactive I_2_ state (**F**). The centrality profiles are shown in blue lines and ABL positions G269, Y272, M309L, L320I, T334, F378, L389, F401, T408Y and H415P are shown in yellow-colored filled circles. Note that these sites are associated with the broader spectrum of experimentally known Imatinib-resistant ABL mutations G269E, Y272H, M309L, L320I, T334I, F378V, L389M, F401L, T408Y, and H415P [30].

Using the ensemble-averaged model of the residue interaction networks, we computed several fundamental network properties: residue betweenness (or residue centrality) and edge betweenness. Different from previous implementations of the allosteric networks, here we employed the AF2-generated conformational ensembles of the active and inactive forms obtained for ABL mutants with alanine scanning adaptation of AF2. A global network parameter, residue centrality, was used to construct the distribution profile and identify key mediating hotspots of allosteric interaction networks that are assumed to correspond to the profile peaks (Figure 10A–C). We defined the residues with a Z-score ≥2 (see Materials and Methods) as high centrality sites. These positions correspond to the highest centrality distribution peaks and are considered to be potential allosteric hotspots.

Although there are obvious similarities between the residue centrality distributions for the ABL states, we also noticed important differences. First, all three network centrality profiles are characterized by multiple peaks that are distributed across the entire kinase domain. However, major allosteric clusters are associated with the regulatory αC-helix (residues 291–311), the αF-helix (residues 436–453), R-spine (M309, L320, F401), 400-DFG-402 motif and 424-WTAPE-428 motif that anchors the substrate binding P+1 loop to the αF-helix, providing a plausible route for signal communication to allosteric binding (Figure 10A–C). It is well known that along with αC-helix being involved in coordination of structural changes, the αF-helix is a central scaffold for assembly of the entire molecule where the R-spine and C-spine anchor all the elements important for catalysis [23,24,25,26]. Despite dramatic structural differences between the active and inactive states, allosteric networks may utilize the αF-helix and αC-helix along with the R-spine for mediating efficient long-range communications. At the same time, the centrality in the active state and intermediate I1 form showed only small peaks for C-lobe residues (Figure 10A,B) while C-lobe regions become engaged in allosteric networks in the I2 form (Figure 10C).

We also examined the relationship between predicted network centrality sites and positions that are associated with the spectrum of experimentally known Imatinib-resistant ABL mutations G269E, Y272H, M309L, L320I, T334I, F378V, L389M, F401L, T408Y, and H415P [30]. By mapping mutational sites onto the centrality profiles, we noticed that M309, L320, and T334 mutational positions along with F401 of the DFG motif that are fundamentally important for assembly and stabilization of the R-spine coincide with the highest centrality peaks in the active and intermediate I forms, thus corresponding to critical allosteric hotspots of long-range interactions (Figure 10A,B). Other mutational positions G269E in the P-loop as well as T408Y and H415P in the open A-loop have low centrality values and are not mediating sites of allosteric networks. These positions are located in flexible regions and can be dynamically coupled with the major allosteric mediating hotspots in the global interaction network connecting the αC-helix-in, the R-spine and the substrate binding site in the active state. In network terms, the emergence of various high centrality sites that are spatially distributed typically implies a broad allosteric network with distinct communication paths connecting functional regions. A strong allosteric network dependency on these mediating sites may explain the sensitivity of the state equilibrium to M309L and L320I mutations in the R-spine leading to a shift from the active to fully closed inactive structure. A noticeably different distribution of the mapped mutational position is seen in the in the I_2_ state where allosteric contributions of M309L and L320I become depleted due to disassembly of the R-spine and only T334I and F401 sites remain dominant centrality peaks (Figure 10C). In contrast to the active ABL form, we observed other mediating sites corresponding to residues Y408, Y412, L448, I451, T453, Y454 as well as C-spine residues C388, L389, and I451. The NMR analysis showed a significant role of Y408 in increasing the population of the I_2_ state, and the interactions of the displaced Y412 A-loop residue with the L403/L406/M407 positions are important in allosteric changes of the inactive I_2_ form.

**Figure 11:**
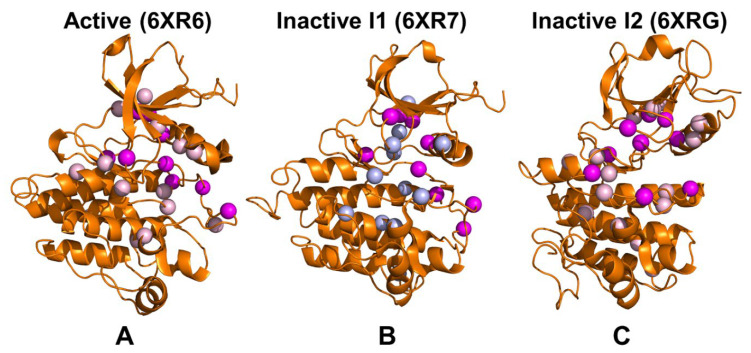
Structural mapping of network-mediating allosteric sites in the active ABL (**A**), inactive I_1_ state (**B**) and inactive I_2_ state (**C**). The kinase domain is shown in orange ribbons and the predicted allosteric hotspots are in grey-colored spheres. The ABL positions G269, Y272, M309L, L320I, T334, F378, L389, F401, T408Y, and H415P are shown in magenta spheres.

Using a community decomposition method, the residue interaction networks were divided into local modules in which residue nodes are strongly interconnected through dynamic correlations, whereas residues that belong to different communities may be only weakly coupled. To characterize global bridges from a community structure, the community bridgeness metric was computed (Figure 10D–F). The network bridgeness profiles showed the increased modularity in the inactive forms manifested in the greater number of peaks as compared to the active state (Figure 10E,F). Among major network bridgeness peaks are M309L, L320I and F401 sites that engage in transmitting allosteric signals and connecting structural clusters in the network. A number of studied ABL mutants, including P-loop mutations G269E, Y272H, A-loop mutation H415P and gate-keeper T334I, are recognized as important Imatinib-resistance mutations even though some of these positions are far away from the inhibitor. Analysis of the interaction networks has shown that residue centrality can provide insight into an allosteric role of Imatinib drug resistance mutations. The results of network analysis showed that the T334 residue is the dominant high centrality position in the network distributions and therefore may correspond to the important allosteric hotspot that controls long-range communications in the global interaction network (Figure 10 and Figure 11). These observations are consistent with the NMR experimental discoveries that allosteric mutations, and most notably T334I mutation, can divert the equilibrium from the conformational state to which Imatinib selectively binds and incurs dug resistance by shifting the equilibrium between the inactive and active forms, which is the fundamental signature of the allosteric kinase mutant [29,30].

From the network-centric perspective, mutations in high centrality positions M309L, L320I, and T334I could perturb R-spine assembly, affect the long-range interactions and alter the global interaction network which may explain the state-switching impact of these modifications. We suggest that sensitivity of the ABL interaction networks to targeted perturbations of these highly connected hot spots may explain why mutation of T334 residue and P-loop mutations could leverage a local perturbation to switch the conformational equilibrium between the inactive and active forms. Structural mapping of the predicted allosteric sites that mediate long-range interactions as well as sites of ABL mutations showed that mutational positions either directly match with the allosteric hotspots or residue within 5 Å distance from these allosteric sites (Figure 11). Indeed, mutational positions G269, Y272, M309L, L320I, T334, F378, L389, and F401 correspond to the predicted allosteric sites that are characterized by the high network centrality. Only the peripheral A-loop residues T408 and H415, which are associated with the experimentally known allosteric mutations T408Y and H415P, do not directly overlap with the allosteric hotspots but are rather located within a close proximity (<5 Å distance) from the predicted allosteric sites (Figure 11). The allosteric networks are more broadly distributed in the active form (Figure 11A) and intermediate state (Figure 11B) connecting the R-spine with the highly dynamic A-loop and substrate binding site. Interestingly, structural rearrangements observed in the inactive I_2_ form produce several local clusters of allosteric hotspots that also include all mutational positions (Figure 11C). As a result, a more localized and narrow interaction network may be a signature of the closed inactive I_2_ state (Figure 11C) that engages a significant fraction of the A-loop residues in allosteric clusters and becomes highly dependent on all mutational sites that are allosterically coupled and function as switches of the allosteric states.

To conclude, the network-based allosteric analysis of the AF2-generated ensembles suggested that functional ABL mutations can directly target or be structurally proximal to allosteric hotpots of long-range interactions. The network analysis suggests that manipulation of attention mechanism to allow for recognizing long-range dependencies between sequence positions may be helpful to detect mutation-induced structural transformations.

## 3. Materials and Methods

### 3.1. MSA Shallow Subsampling Adaptation of AF2

Structural prediction of the ABL kinase states were conducted using the AF2 framework [1,2] within the ColabFold [42] using a range of MSA depths and MSA subsampling. The MSAs were generated using the MMSeqs2 library [43] using the ABL1 sequence from residues 240 to 440 as input. We used max_msa field in the ColabFold to set two AF2 parameters in the following format: max_seqs:extra_seqs. These parameters determine the number of sequences subsampled from the MSA where max_seqs sets the number of sequences passed to the row/column attention track and extra_seqs is the number of sequences additionally processed by the main evoformer stack. The default MSAs are subsampled randomly to obtain shallow MSAs containing as few as five sequences. We ran simulations with max_seqs:extra_seqs 16:32, 32:64, 64:128. 128:256, 256:512 and 512:1024 values and report the results at max_seqs:extra_seqs 16:32 that produced the greatest diversity. The lower values encourage more diverse predictions but increase the number of misfolded models. We additionally manipulated the num_recycles parameters to produce more diverse outputs. To generate more data, we set num_recycles to 12, which produces 14 structures starting from recycle 0 to recycle 12 and generates a final refined structure. Recycling is an iterative refinement process, with each recycled structure becoming more precise. AF2 makes predictions using 5 models pretrained with different parameters, and consequently with different weights. Each of these models generates 14 structures, amounting to 70 structures in total. We then set the num_seed parameter to 1. This parameter quantifies the number of random seeds to iterate through, ranging from random_seed to random_seed+num_seed. We also enabled the use_dropout parameter, meaning that dropout layers in the model would be active during the time of predictions.

### 3.2. AF2 with Randomized Alanine Sequence Scanning and Shallow Subsampling

The initial input for the full sequence randomized alanine scanning is the original full native sequence. This technique utilizes an algorithm that iterates through each amino acid in the native sequence and randomly substitutes 5–15% of the residues with alanine, to simulate random alanine substitution mutations [35]. The algorithm substitutes residue with alanine at each position with a probability randomly generated between 0.05 and 0.15 for each sequence position. We ran this algorithm multiple times (~10–50) on the full sequences for each mutant, resulting in a multitude of distinct sequences, each with different frequency and position of alanine mutations. MSAs are then constructed for each of these mutated sequences using the alanine-scanned full-length sequences as input for the MMSeqs2 program [43]. The AF2 shallow MSA methodology is subsequently employed on these MSAs to predict protein structures as described previously. A total of 70 predicted structures were generated from 12 recycles per model. In addition to randomized alanine sequence scanning of the complete sequence, we also examined several variations of this approach with targeted alanine masking of the ABL sequence space. In particular, we probed the effects of random alanine masking of sequence positions in the A-loop (residues 398–421) that is critical for conformational change between the active and inactive ABL forms. For each of these targeted alanine making experiments, we generate 10 alanine scanned sequences, each with different frequency and position of alanine mutations in the respective A-loop and C-terminal loop regions.

AF2 models were ranked by Local Distance Difference Test (pLDDT) scores (a per-residue estimate of the prediction confidence on a scale from 0 to 100), quantified by the fraction of predicted Cα distances that lie within their expected intervals. The values correspond to the model’s predicted scores based on the lDDT-Cα metric which is a local superposition-free metric that assesses the atomic displacements of the residues in the predicted model [1,2]. Models ranked in the top five were compared to the experimental structure using a TM-score which is a metric for assessing the topological similarity of protein structures based on their given residue equivalency [44,45]. Notably this tool is not applied to compare two proteins of different sequences. The TM-score gives greater emphasis to smaller distance errors compared to larger ones, making it more sensitive to global fold similarity rather than local structural variations. Additionally, it uses a length-dependent scaling to normalize distance errors, ensuring that the magnitude of the TM-score remains independent of protein length when comparing random structure pairs [44,45]. The TM-score ranges from 0 to 1, where a value of 1 indicates a perfect match between the predicted model and the reference structure. When the TM-score > 0.5, it implies that the structures share roughly the same fold. A TM-score > 0.5 is often used as a threshold to determine if the predicted model has a fold similar to the reference structure. If the TM-score is above this threshold, it suggests that the predicted structure and the reference structure have a significant structural resemblance [45]. The distributions of TM-scores were calculated in terms of the averages of the highest TM-scores for AF2 models compared to PDB structures of the same kinases for each MSA depth and structural conformation.

### 3.3. Principal Component Analysis (PCA)

Principal Component Analysis (PCA) is a dimensionality reduction method that is often used to analyze protein structures and protein conformational ensembles by reducing variation observed within 3D atomic coordinates of the protein structures. In this study, PCA is used to project the correlated motions of the ABL kinase domain residues onto a set of linearly uncorrelated variables’ principal components for both the NMR ensembles of the ABL states and AF2-generated ensembles of the ABL WT and ABL double mutants. This method is based on the construction of the covariance matrix of the coordinate fluctuations of the simulated proteins. The eigenvectors are the principal components that represent the directions of the coordinated motions of atoms. The eigenvalues indicate the magnitude of the motions along the movement direction. The eigenvectors and eigenvalues are obtained by diagonalizing the covariance matrix, which provides information about correlated motions throughout the protein. PCA was performed to probe the difference in internal dynamics and conformational changes of the ABL WT states and examined ABL double mutants as well as to compare how mutation-induced dynamic changes can alter the preferential population of the active and inactive ABL states.

PCA analysis was performed by merging the NMR ensembles of the ABL WT and AF2-generated ensembles for ABL WT and double mutants and projecting the PCA maps onto common principal components of the ABL WT protein. Using the projected principal components (eigenvectors) of the ABL WT as the common reference coordinate system, we used the merged ensemble to produce the PCA maps for the AF2-generated ensembles that can be directly compared to the PCAs of the experimentally determined NMR ensemble as the points are projected and plotted in the same coordinate system. Python package ProDy was used to perform PCA for C-alpha cartesian atoms for the systems [46]. This program allows for characterization of structural variations in heterogeneous datasets of NMR structures as well as for comparison of these variations with the computationally generated equilibrium dynamics ensembles using MD simulations or AF2-generated protein conformations. The AF2-generated models with pLDDT < 70 were excluded from PCA computations to remove a bias of partially misfolded or disordered conformational states. For additional validation and comparison, PCA of the merged ensemble derived from NMR ABL WT structures and AF2-generated ensembles for ABL double mutants was also conducted using the Bio3D package [47] revealing the same motion patterns of the protein systems as predicted by ProDy [46]. The reported PCA plots are obtained using the ProDy program [46].

### 3.4. Protein Structure Network Analysis

A graph-based representation of protein structures [48] is used to represent residues as network nodes and the inter-residue edges to describe non-covalent residue interactions. The network edges that define residue connectivity are based on non-covalent interactions between residue sidechains. The weights of the network edges in the residue interaction networks are determined by dynamic residue cross-correlations obtained from AF2-generated conformational ensembles [49,50,51,52]. The residue interaction networks were constructed by incorporating the residue connectivity and residues cross-correlations. The edge lengths in the network are obtained using the generalized correlation coefficients $R_{⬚}\left(X_{i},X_{j}\right)$ that characterize the dynamic correlation for a pair of protein residues. The length (i.e., weight) $w_{ij}=-\log\left[R_{⬚}\left(X_{i},X_{j}\right)\right]$ of the edge that connects nodes i and j is defined as the element of a matrix measuring the correlation coefficient $R_{⬚}\left(X_{i},X_{j}\right)$ as between residue fluctuations. We employed the generalized correlation (GC) coefficient first proposed by Lange and Grubmüller [53] that measures the degree of correlation between Cα atoms based on their mutual information. The correlation tool in the Gromacs 3.3 package was used [54]. A similar strategy for analysis of allosteric motions and interactions was successfully undertaken and improved in a series of studies [55,56] where the introduced GC matrix proved to be a sensitive and accurate method for detecting the interdependence of spatially distant residues, providing a reliable and reproducible measure of how much the motion of one residue is dependent on the fluctuations of another spatially separated residue. In this model, highly correlated pairs of residues are associated with efficient links for information communication on the residue interaction network. Similar to these studies, during the construction of the residue interaction network, each node pair was connected by an edge if the respective residues remain within 5 Å for at least 75% of generated conformations within a given ensemble. Using these assumptions, we can effectively build a dynamic residue interaction network that is obtained from AF2-generated ensembles of structures. The ensemble of the shortest paths is determined from matrix of communication distances by the Floyd–Warshall algorithm [57]. Network graph calculations were performed using the python package NetworkX [58]. Using the constructed protein structure networks, we computed the residue-based betweenness parameter. The betweenness of residue i is defined to be the sum of the fraction of shortest paths between all pairs of residues that pass through residue i:

(2) $$C_{b}(n_{i})=\sum_{j<k}^{N}\frac{g_{jk}(i)}{g_{jk}}$$

where $g_{jk}$ denotes the number of shortest geodesics paths connecting j and *k*, and $g_{jk}(i)$ is the number of shortest paths between residues j and *k* passing through the node $n_{i}$. For each residue, a Z-score per node was calculated where *k* is a node, C is the centrality, $\bar{C}$ is its average, and σ is the corresponding standard deviation. The Z-score is defined as follows:

(3) $$Z_{k}=\frac{C_{k}-\bar{C}}{\sigma }$$

We define the residues with a Z-score ≥ 2 as high centrality positions which typically correspond to the highest centrality distribution peaks and are considered to be potential allosteric hotspots in our analysis.

The Girvan–Newman algorithm [59,60,61] is used to identify local communities and optimize modularity of the interaction network. In this approach, edge centrality (edge betweenness) is defined as the ratio of all the shortest paths passing through a particular edge to the total number of shortest paths in the network. To characterize global bridges from a community structure, we introduce a community bridgeness metric similar to Rao–Stirling index [62,63,64]. This parameter uses as input a prior categorization of the nodes into distinct communities:

(4) $$G\left(i\right)=\sum_{j\in J}l_{IJ}\delta_{iJ}$$

where the sum is over communities J (different from the community of node i, denoted as I), $\delta_{iJ}$ is equal 1 if there is a link between node i and community J and 0 otherwise. $l_{iJ}$ corresponds to the effective distance between community I and community J as measured by the inverse of the number of links between them. All topological measures were computed using the python module, the python package NetworkX [58], and also the Cytoscape platform for network analysis [65]. The ModuLand program within the Cytoscape platform [66,67] was also adapted to determine a hierarchical network structure and compute residue bridgeness of the identified communities in the dynamic residue interaction networks. The RING program [68,69] was used to generate the initial residue interaction networks.

## 4. Conclusions

In the current study, we examined several AF2 adaptations to characterize conformational ensembles and populations for a panel of experimentally known state-switching double ABL mutants. The results of our analysis showed that widely used shallow MSA subsampling can characterize conformational heterogeneity of the ABL active form but generally is not robust for predicting mutation-induced structural changes resulting in the inactive ABL states. This study demonstrated that randomized and targeted alanine sequence masking scanning combined with shallow MSA subsampling can significantly expand the conformational diversity of the predicted structural ensembles and predict populations of both active and inactive ABL states. These results are particularly significant as AF2 predictions of allosteric systems including protein kinases are strongly biased towards predictions of the thermodynamically stable ground states and typically fail to detect less stable “excited” states. Our results showed that mutational effects that induce conformational equilibrium switches can be simulated by AF2 adaptations invoking random sequence masking across the entire sequence which is followed by MSA construction and shallow MSA subsampling. Although AF2 predictions can capture functionally relevant conformations, the generated ensembles cannot be directly compared with the thermodynamic equilibrium ensemble of conformations. Hence, using AF2 predictions directly for characterization of the conformational landscape might be challenging since this is not a completely physically correct thermodynamic ensemble of states. MD simulations provide a more detailed and granular view of conformational flexibility and structural changes that are intimately connected with physical models of protein energetics. Our results underscore that learning specific patterns of coevolutionary signals together with allosteric-centric structural information on long-range couplings and attention-based learning of allosteric couplings across homologous folds may augment the predictive abilities of AF2-based methods. AF2 predictions often need to be considered in combination with MD simulations of the predicted multiple conformations to produce the Boltzmann-weighted ensemble may that can characterize the relative energetics and probabilities of different conformational states. Combining the proposed AF2 adaptations showing promise in describing allosteric states and ensembles with subsequent MD simulations initiated from predicted conformations can enable robust characterization of structure and dynamic mechanisms by marrying the accuracy of structural predictions with the details of equilibrium ensembles resulting in a more realistic representation of molecular events [70].

The recent introduction of AlphaFold3 (AF3) in May 2024 which expanded protein structure prediction towards biomolecular complexes of proteins, nucleic acids and their ligands, directly from their sequences, marked another significant step forward towards understanding how biomolecules interact [71]. AF3 displayed superior accuracy in predicting protein–DNA and protein–RNA complexes, and outperformed AlphaFold Multimer in predicting protein–protein complexes [72], attributing the improvements to a new diffusion module, which is trained to directly predict the cartesian coordinates of individual atoms and can be generalized to the chemical space. However, the launch of the AF3 server was not free of controversy, as the release of AF3 lacked the open source code [73]. While AF2 and AlphaFold-Multimer are open-sourced, AF3 remains partially accessible through a limited online server and has not been open-sourced, restricting further development. There are ongoing efforts from the open source community to advance open source biomolecular structure prediction including the recent release of the full AF3 model along with the training code (https://github.com/Ligo-Biosciences/AlphaFold3) (accessed on 29 May 2024). The PaddleHelix research team at Baidu have released their AF3 replication.

HelixFold3 is an open source on GitHub aiming to reproduce AF3 capabilities in predicting the structures of the conventional ligands, nucleic acids, and proteins [74]. In September 2024, a new AI-enabled system termed AlphaProteo was released by the AlphaFold team aiming for the design of novel, high-strength protein binders for diverse target proteins (https://deepmind.google/discover/blog/alphaproteo-generates-novel-proteins-for-biology-and-health-research/) (accessed on 29 May 2024), (Google DeepMind Technologies Limited. London, UK). Despite a flurry of new exciting AI tools, robust predictions of conformational ensembles and mapping of conformational landscapes with multiple functional states from sequences remains a major fundamental problem for AI applications in biology and would require a large dataset of experimentally determined ensembles and potentially improved network architectures—a challenge that is embraced by the rapidly growing community of researchers bringing AI to biomedical and life sciences.

## Data Availability

The original contributions presented in the study are included in the article/supplementary material, further inquiries can be directed to the corresponding author.

## Supplementary Materials

The following supporting information can be downloaded at: https://www.mdpi.com/article/10.3390/ijms251810082/s1.

## References

[1] Jumper J., Evans R., Pritzel A., Green T., Figurnov M., Ronneberger O., Tunyasuvunakool K., Bates R., Žídek A., Potapenko A. (2021). Highly Accurate Protein Structure Prediction with AlphaFold. Nature.

[2] Tunyasuvunakool K., Adler J., Wu Z., Green T., Zielinski M., Žídek A., Bridgland A., Cowie A., Meyer C., Laydon A. (2021). Highly Accurate Protein Structure Prediction for the Human Proteome. Nature.

[3] Bahdanau D., Cho K., Bengio Y. (2014). Neural Machine Translation by Jointly Learning to Align and Translate. arXiv.

[4] Vaswani A., Shazeer N., Parmar N., Uszkoreit J., Jones L., Gomez A., Kaiser L., Polosukhin I. (2017). Attention is all you need. Adv. Neural Inf. Process. Syst..

[5] Rives A., Meier J., Sercu T., Goyal S., Lin Z., Liu J., Guo D., Ott M., Zitnick C.L., Ma J. (2021). Biological Structure and Function Emerge from Scaling Unsupervised Learning to 250 Million Protein Sequences. Proc. Natl. Acad. Sci. USA.

[6] Lin Z., Akin H., Rao R., Hie B., Zhu Z., Lu W., Smetanin N., Verkuil R., Kabeli O., Shmueli Y. (2023). Evolutionary-Scale Prediction of Atomic-Level Protein Structure with a Language Model. Science.

[7] Wu R., Ding F., Wang R., Shen R., Zhang X., Luo S., Su C., Wu Z., Xie Q., Berger B. (2022). High-Resolution de Novo Structure Prediction from Primary Sequence. bioRxiv.

[8] Fleishman S.J., Horovitz A. (2021). Extending the New Generation of Structure Predictors to Account for Dynamics and Allostery. J. Mol. Biol..

[9] Sala D., Engelberger F., Mchaourab H.S., Meiler J. (2023). Modeling Conformational States of Proteins with AlphaFold. Curr. Opin. Struct. Biol..

[10] Yin R., Feng B.Y., Varshney A., Pierce B.G. (2022). Benchmarking AlphaFold for Protein Complex Modeling Reveals Accuracy Determinants. Protein Sci..

[11] Kryshtafovych A., Montelione G.T., Rigden D.J., Mesdaghi S., Karaca E., Moult J. (2023). Breaking the Conformational Ensemble Barrier: Ensemble Structure Modeling Challenges in CASP15. Proteins.

[12] Bret H., Gao J., Zea D.J., Andreani J., Guerois R. (2024). From Interaction Networks to Interfaces, Scanning Intrinsically Disordered Regions Using AlphaFold2. Nat. Commun..

[13] Saldaño T., Escobedo N., Marchetti J., Zea D.J., Mac Donagh J., Velez Rueda A.J., Gonik E., García Melani A., Novomisky Nechcoff J., Salas M.N. (2022). Impact of Protein Conformational Diversity on AlphaFold Predictions. Bioinformatics.

[14] Chakravarty D., Porter L.L. (2022). AlphaFold2 Fails to Predict Protein Fold Switching. Protein Sci..

[15] Ma P., Li D., Brüschweiler R. (2023). Predicting Protein Flexibility with AlphaFold. Proteins.

[16] Versini R., Sritharan S., Aykac Fas B., Tubiana T., Aimeur S.Z., Henri J., Erard M., Nüsse O., Andreani J., Baaden M. (2024). A Perspective on the Prospective Use of AI in Protein Structure Prediction. J. Chem. Inf. Model..

[17] Nussinov R., Zhang M., Liu Y., Jang H. (2022). AlphaFold, Artificial Intelligence (AI), and Allostery. J. Phys. Chem. B.

[18] Del Alamo D., Sala D., Mchaourab H.S., Meiler J. (2022). Sampling Alternative Conformational States of Transporters and Receptors with AlphaFold2. eLife.

[19] Stein R.A., Mchaourab H.S. (2022). SPEACH_AF: Sampling Protein Ensembles and Conformational Heterogeneity with Alphafold2. PLoS Comput. Biol..

[20] Wayment-Steele H.K., Ovchinnikov S., Colwell L., Kern D. (2024). Predicting multiple conformations via sequence clustering and AlphaFold2. Nature.

[21] Sala D., Hildebrand P.W., Meiler J. (2023). Biasing AlphaFold2 to Predict GPCRs and Kinases with User-Defined Functional or Structural Properties. Front. Mol. Biosci..

[22] Yang Z., Zeng X., Zhao Y., Chen R. (2023). AlphaFold2 and Its Applications in the Fields of Biology and Medicine. Signal Transduct. Target. Ther..

[23] Taylor S.S., Kornev A.P. (2011). Protein Kinases: Evolution of Dynamic Regulatory Proteins. Trends Biochem. Sci..

[24] Taylor S.S., Wu J., Bruystens J.G.H., Del Rio J.C., Lu T.-W., Kornev A.P., Ten Eyck L.F. (2021). From Structure to the Dynamic Regulation of a Molecular Switch: A Journey over 3 Decades. J. Biol. Chem..

[25] Johnson T.K., Bochar D.A., Vandecan N.M., Furtado J., Agius M.P., Phadke S., Soellner M.B. (2021). Synergy and Antagonism between Allosteric and Active-Site Inhibitors of Abl Tyrosine Kinase. Angew. Chem. Int. Ed..

[26] Kim C., Ludewig H., Hadzipasic A., Kutter S., Nguyen V., Kern D. (2023). A Biophysical Framework for Double-Drugging Kinases. Proc. Natl. Acad. Sci. USA.

[27] Paul F., Thomas T., Roux B. (2020). Diversity of Long-Lived Intermediates along the Binding Pathway of Imatinib to Abl Kinase Revealed by MD Simulations. J. Chem. Theory Comput..

[28] Paul F., Meng Y., Roux B. (2020). Identification of Druggable Kinase Target Conformations Using Markov Model Metastable States Analysis of Apo-Abl. J. Chem. Theory Comput..

[29] Saleh T., Rossi P., Kalodimos C.G. (2017). Atomic View of the Energy Landscape in the Allosteric Regulation of Abl Kinase. Nat. Struct. Mol. Biol..

[30] Xie T., Saleh T., Rossi P., Kalodimos C.G. (2020). Conformational states dynamically populated by a kinase determine its function. Science.

[31] Krishnan K., Tian H., Tao P., Verkhivker G.M. (2022). Probing Conformational Landscapes and Mechanisms of Allosteric Communication in the Functional States of the ABL Kinase Domain Using Multiscale Simulations and Network-Based Mutational Profiling of Allosteric Residue Potentials. J. Chem. Phys..

[32] Faezov B., Dunbrack R.L. (2023). AlphaFold2 Models of the Active Form of All 437 Catalytically Competent Human Protein Kinase Domains. bioRxiv.

[33] Herrington N.B., Stein D., Li Y.C., Pandey G., Schlessinger A. (2023). Exploring the Druggable Conformational Space of Protein Kinases Using AI-Generated Structures. bioRxiv.

[34] Monteiro da Silva G., Cui J.Y., Dalgarno D.C., Lisi G.P., Rubenstein B.M. (2024). High-Throughput Prediction of Protein Conformational Distributions with Subsampled AlphaFold2. Nat. Commun..

[35] Raisinghani N., Alshahrani M., Gupta G., Tian H., Xiao S., Tao P., Verkhivker G. (2024). Interpretable Atomistic Prediction and Functional Analysis of Conformational Ensembles and Allosteric States in Protein Kinases Using AlphaFold2 Adaptation with Randomized Sequence Scanning and Local Frustration Profiling. bioRxiv.

[36] Wang L., Wen Z., Liu S.-W., Zhang L., Finley C., Lee H.-J., Fan H.-J.S. (2024). Overview of AlphaFold2 and breakthroughs in overcoming its limitations. Comput. Biol. Med..

[37] Buel G.R., Walters K.J. (2022). Can AlphaFold2 Predict the Impact of Missense Mutations on Structure?. Nat. Struct. Mol. Biol..

[38] Pak M.A., Markhieva K.A., Novikova M.S., Petrov D.S., Vorobyev I.S., Maksimova E.S., Kondrashov F.A., Ivankov D.N. (2023). Using AlphaFold to Predict the Impact of Single Mutations on Protein Stability and Function. PLoS ONE.

[39] Stein R.A., Mchaourab H.S. (2024). Rosetta Energy Analysis of AlphaFold2 Models: Point Mutations and Conformational Ensembles. bioRxiv.

[40] McBride J.M., Polev K., Abdirasulov A., Reinharz V., Grzybowski B.A., Tlusty T. (2023). AlphaFold2 Can Predict Single-Mutation Effects. Phys. Rev. Lett..

[41] McBride J.M., Tlusty T. (2024). AI-Predicted Protein Deformation Encodes Energy Landscape Perturbation. Phys. Rev. Lett..

[42] Mirdita M., Schütze K., Moriwaki Y., Heo L., Ovchinnikov S., Steinegger M. (2022). ColabFold: Making Protein Folding Accessible to All. Nat. Methods.

[43] Steinegger M., Söding J. (2017). MMseqs2 Enables Sensitive Protein Sequence Searching for the Analysis of Massive Data Sets. Nat. Biotechnol..

[44] Zhang Y., Skolnick J. (2004). Scoring Function for Automated Assessment of Protein Structure Template Quality. Proteins.

[45] Xu J., Zhang Y. (2010). How Significant Is a Protein Structure Similarity with TM-Score = 0.5?. Bioinformatics.

[46] Bakan A., Meireles L.M., Bahar I. (2011). ProDy: Protein Dynamics Inferred from Theory and Experiments. Bioinformatics.

[47] Grant B.J., Rodrigues A.P.C., ElSawy K.M., McCammon J.A., Caves L.S.D. (2006). Bio3d: An R Package for the Comparative Analysis of Protein Structures. Bioinformatics.

[48] Vijayabaskar M.S., Vishveshwara S. (2010). Interaction energy based protein structure networks. Biophys. J..

[49] Sethi A., Eargle J., Black A.A., Luthey-Schulten Z. (2009). Dynamical networks in tRNA:protein complexes. Proc. Natl. Acad. Sci. USA.

[50] Stetz G., Verkhivker G.M. (2017). Computational analysis of residue interaction networks and coevolutionary relationships in the Hsp70 chaperones: A community-hopping model of allosteric regulation and communication. PLoS Comput. Biol..

[51] Astl L., Verkhivker G.M. (2019). Atomistic Modeling of the ABL Kinase Regulation by Allosteric Modulators Using Structural Perturbation Analysis and Community-Based Network Reconstruction of Allosteric Communications. J. Chem. Theory Comput..

[52] Verkhivker G.M., Agajanian S., Oztas D.Y., Gupta G. (2021). Comparative Perturbation-Based Modeling of the SARS-CoV-2 Spike Protein Binding with Host Receptor and Neutralizing Antibodies: Structurally Adaptable Allosteric Communication Hotspots Define Spike Sites Targeted by Global Circulating Mutations. Biochemistry.

[53] Lange O.F., Grubmuller H. (2006). Generalized Correlation for Biomolecular Dynamics. Proteins.

[54] Lindahl E., Hess B., Van Der Spoel D. (2001). GROMACS 3.0: A package for molecular simulation and trajectory analysis. J. Mol. Model..

[55] East K.W., Newton J.C., Morzan U.N., Narkhede Y.B., Acharya A., Skeens E., Jogl G., Batista V.S., Palermo G., Lisi G.P. (2020). Allosteric Motions of the CRISPR-Cas9 HNH Nuclease Probed by NMR and Molecular Dynamics. J. Am. Chem. Soc..

[56] Nierzwicki Ł., Arantes P.R., Saha A., Palermo G. (2021). Establishing the allosteric mechanism in CRISPR-Cas9. WIREs Comput. Mol. Sci..

[57] Floyd R.W. (1962). Algorithm 97: Shortest Path. Commun. ACM.

[58] Hagberg A., Schult D.A., Swart P.J., Varoquaux G., Vaught T., Millman J. Exploring Network Structure, Dynamics, and Function using NetworkX. Proceedings of the 7th Python in Science Conference (SciPy2008).

[59] Girvan M., Newman M.E. (2002). Community Structure in Social and Biological Networks. Proc. Natl. Acad. Sci. USA.

[60] Newman M.E., Girvan M. (2004). Finding and Evaluating Community Structure in Networks. Phys. Rev. E.

[61] Newman M.E. (2006). Modularity and Community Structure in Networks. Proc. Natl. Acad. Sci. USA.

[62] Jensen P., Morini M., Karsai M., Venturini T., Vespignani A., Jacomy M., Cointet J.-P., Mercklé P., Fleury E. (2016). Detecting Global Bridges in Networks. J. Complex Netw..

[63] Rao C.R. (1982). Diversity and Dissimilarity Coefficients: A Unified Approach. Theor. Popul. Biol..

[64] Stirling A. (2007). A general Framework for Analyzing Diversity in Science, Technology and Society. J. R. Soc. Interface.

[65] Shannon P., Markiel A., Ozier O., Baliga N.S., Wang J.T., Ramage D., Amin N., Schwikowski B., Ideker T. (2003). Cytoscape: A software environment for integrated models of biomolecular interaction networks. Genome Res..

[66] Kovács I.A., Palotai R., Szalay M.S., Csermely P. (2010). Community landscapes: An integrative approach to determine overlapping network module hierarchy, identify key nodes and predict network dynamics. PLoS ONE.

[67] Szalay-Beko M., Palotai R., Szappanos B., Kovacs I.A., Papp B., Csermely P. (2012). ModuLand plug-in for Cytoscape: Determination of hierarchical layers of overlapping network modules and community centrality. Bioinformatics.

[68] Clementel D., Del Conte A., Monzon A.M., Camagni G.F., Minervini G., Piovesan D., Tosatto S.C.E. (2022). RING 3.0: Fast Generation of Probabilistic Residue Interaction Networks from Structural Ensembles. Nucleic Acids Res..

[69] Del Conte A., Camagni G.F., Clementel D., Minervini G., Monzon A.M., Ferrari C., Piovesan D., Tosatto S.C.E. (2024). RING 4.0: Faster Residue Interaction Networks with Novel Interaction Types across over 35,000 Different Chemical Structures. Nucleic Acids Res..

[70] Brown B.P., Stein R.A., Meiler J., Mchaourab H.S. (2024). Approximating Projections of Conformational Boltzmann Distributions with AlphaFold2 Predictions: Opportunities and Limitations. J. Chem. Theory Comput..

[71] Abramson J., Adler J., Dunger J., Evans R., Green T., Pritzel A., Ronneberger O., Willmore L., Ballard A.J., Bambrick J. (2024). Accurate Structure Prediction of Biomolecular Interactions with AlphaFold 3. Nature.

[72] Roy R., Al-Hashimi H.M. (2024). AlphaFold3 Takes a Step toward Decoding Molecular Behavior and Biological Computation. Nat. Struct. Mol. Biol..

[73] Callaway E. (2024). Who Will Make AlphaFold3 Open Source? Scientists Race to Crack AI Model. Nature.

[74] Liu L., Zhang S., Xue Y., Ye X., Zhu K., Li Y., Liu Y., Zhang X., Fang X. (2024). Technical Report of HelixFold3 for Biomolecular Structure Prediction. arXiv.

