# Supplementary material for: Predicting Mutation-Induced Allosteric Changes in Structures and Conformational Ensembles of the ABL Kinase Using AlphaFold2 Adaptations with Alanine Sequence Scanning
Source: Int J Mol Sci. 2024 Sep 19;25(18):10082. doi: 10.3390/ijms251810082 (PMC11432724; doi:10.3390/ijms251810082)
Supplement: Supplementary file 1 [file ijms-25-10082-s001.zip › ijms-3130280-supplementary.pdf]

Supplementary Materials

Predicting Mutation-Induced Allosteric Changes in Structures and Conformational Ensembles of the ABL Kinase Using AlphaFold2 Adaptations with Alanine Sequence Scanning

Nishank Raisinghani [1], Mohammed Alshahrani [1], Grace Gupta [1] and Gennady Verkhivker [1,2,*]

[1] Keck Center for Science and Engineering, Schmid College of Science and Technology, Chapman University, Orange, CA 92866, USA; nishankr@stanford.edu (N.R.); alshahrani@chapman.edu (M.A.); grgupta@chapman.edu (G.G.)
[2] Department of Biomedical and Pharmaceutical Sciences, Chapman University School of Pharmacy, Irvine, CA 92618, USA
* Correspondence: verkhivk@chapman.edu; Tel.: +1-714-516-4586

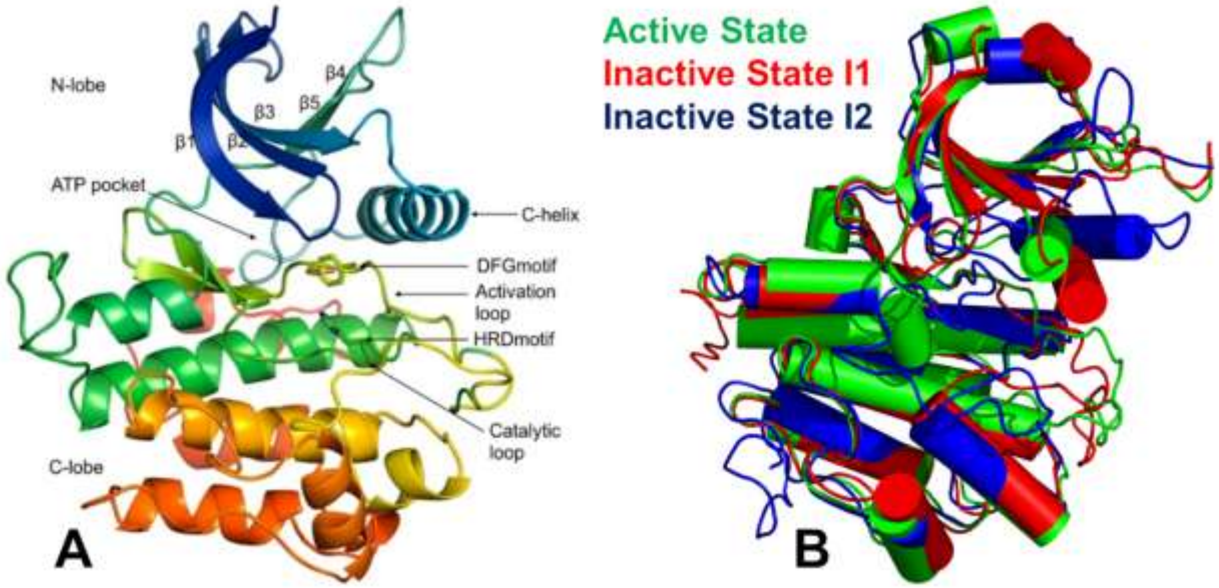

Figure S1. (A) Structural overview of the kinase domain with annotation of the functional regions. (B) Structural superposition of the ensemble-averaged conformations for the active state (in green ribbons), for the inactive I_1 state (in red ribbons), and for the inactive I_2 state (in blue ribbons).

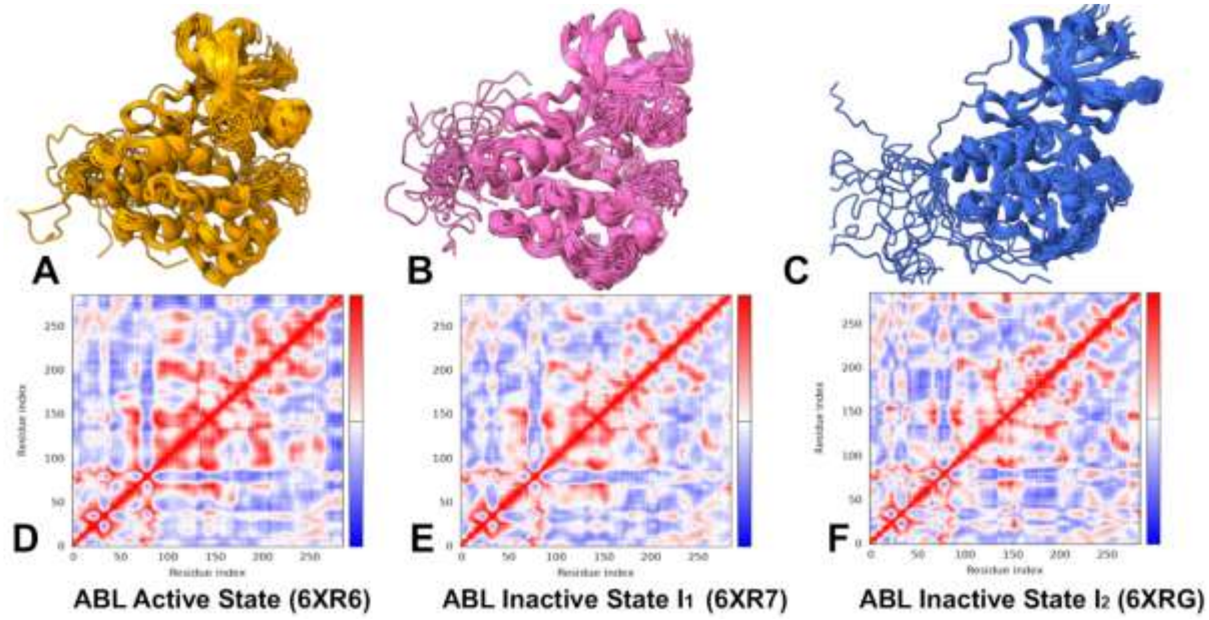

Figure S2. NMR solution structural ensembles of the thermodynamically stable fully active ground state of the ABL kinase domain (pdb id 6XR6) (A), the inactive state I_1 (pdb id 6XR7) (B) and the closed inactive state I_2 (pdb id 6XRG) (C). The covariance maps of dynamic cross-correlations between pairs of residues in the ABL active state (D), the inactive state I_1 (E) and the inactive state I_2 (F). Cross-correlations of residue-based fluctuations vary between +1 (correlated motion; fluctuation vectors in the same direction, colored in dark red) and -1 (anti-correlated motions; fluctuation vectors in the same direction, colored in dark blue). The values > 0.5 are colored in dark red and the lower bound in the color bar indicates the value of the most anti-correlated pairs. Note that the residue numbering in the pdb (residues 248-534) is mapped onto residues 1-286 on panels D-F.
